# From Womb to World: Unravelling the Microbiota′s Impact on Health and Disease

**DOI:** 10.1155/ijm/9228548

**Published:** 2026-07-18

**Authors:** Julio A. Poterico, Babu G. Venkatesh, Luis Jaramillo-Valverde, César Sanchez, Erick Alayo, Julio Valdivia-Silva, Heinner Guio

**Affiliations:** ^1^ INBIOMEDIC Research and Technological Centre, Lima 15046, Peru; ^2^ Escuela de Posgrado, Universidad San Ignacio de Loyola, Lima, Peru, usil.edu.pe; ^3^ Faculty of Health Sciences, Universidad de Huánuco, Huánuco 10001, Peru; ^4^ School of Medicine, Universidad Continental, Huancayo, Peru, ucontinental.edu.pe; ^5^ Laboratorio de Biotecnología y Biología Molecular, Instituto Nacional de Salud, Lima 15046, Peru, ins.gob.pe; ^6^ Division of Gastroenterology, John H. Stroger Hospital of Cook County and Rush Medical College, Chicago, Illinois, USA; ^7^ Centro de Investigación en Bioingeniería, Universidad de Ingeniería y Tecnología (UTEC), Lima 15063, Peru, utec.edu.pe

**Keywords:** human cells, immune responses, microbiota, susceptibility diseases

## Abstract

The human body hosts an intricate array of microbial communities, outnumbering eukaryotic cells. Delving into the nuanced interplay between the microbiota and human cells is essential for comprehending health homeostasis and disease progression. Notably, the microbiota′s influence commences even before birth, sculpting immune responses and delineating susceptibility to chronic diseases in later stages of life. Throughout the course of human life, variations in microbial composition have been observed to correlate with a wide range of pathologies, prompting the development of pioneering treatments aimed at reinstating a healthier microbial balance. Within this narrative review, we expound upon the fundamental role of the microbiota in the continuum of health and disease. Our endeavour is to contribute to a nuanced comprehension of diseases and the development of targeted interventions for their judicious management.

## 1. Introduction

The human body and its gastrointestinal tract are colonised by 10^14^ endogenous microbial communities including bacteria, viruses, fungi and protozoa (most of which are anaerobic bacterial species) [1]; this means that only 10% of the cells belong to the human being. The whole community of microorganisms is known as a *microbiota*, and the amalgamation of their genomic information is referred to as the *microbiome*. In recent decades, advancements in human microbiome research have unravelled its profound influence on human physiology, paving the way for personalised or precision medicine [2]. Notably, substantial disparities in gut microbial composition have been observed between healthy and unhealthy individuals, underscoring the pivotal role of the microbiome in shaping human health [3, 4]. Investigating the origins and implications of this microbial variation is crucial for understanding the microbiome′s impact on the onset and progression of diseases, thereby facilitating precision medicine approaches.

Recent advances in sequencing technologies and bioinformatics methodologies have provided innovative avenues for research. These tools enable the exploration of mechanistic interactions between the host and gut microbiota, shedding light on the significant influence of the gut microbiome on maintaining human physiological well‐being [5]. However, the burgeoning volume of sequencing data generated by gut microbiota projects, employing massive parallel sequencing technologies, coupled with the availability of advanced sequencing data analysis pipelines, has posed a challenge in interpreting the results [6].

Initially, the gut microbiome research advanced through DNA‐based 16S rRNA gene sequencing and shotgun metagenomic sequencing, enabling the annotation of microbiome taxonomic profiles and their functional potential. Recently, metatranscriptomics, an RNA‐based approach, has emerged, offering insights into the active transcripts within a microbiome under specific environmental conditions [7]. This approach identifies crucial active pathways and assesses how expressed functions may impact disease progression and severity [8, 9].

In this manuscript, we delve into the influences of prenatal and neonatal microbial exposure on innate and adaptive immune responses. Additionally, we explore the significant associations of the gut microbiome with disease pathogenesis and discuss the clinical applications of gut microbiota in managing or preventing specific illnesses and disorders. By synthesising the latest advancements in microbiome research, this review is aimed at contributing to the evolving field of precision medicine and fostering a deeper understanding of the intricate interplay between microbial communities and human health.

## 2. The Microbiota in the Prenatal Period: Influences on Immune Responses and Disease Predisposition

For many decades, the intrauterine environment was considered sterile, under the ‘sterile womb hypothesis,’ which proposed that microbial colonisation begins at birth. Advances in sequencing technologies have challenged this view by detecting bacterial DNA in placental tissue, amniotic fluid, umbilical cord blood and meconium, suggesting the possibility of limited in utero microbial exposure. However, this interpretation remains controversial, as low‐biomass samples are highly susceptible to contamination, and consistent evidence for a viable resident placental microbiota is lacking. Despite this ongoing debate, there is growing evidence that maternal microbiota‐derived metabolites, microbial components and immune mediators can cross the placenta and influence fetal immune development. Experimental studies using germ‐free animal models demonstrate that maternal microbial signals contribute to immune maturation in offspring even in the absence of direct fetal colonisation. Overall, current evidence more strongly supports the concept of prenatal microbial influence through maternal signalling rather than definitive in utero colonisation. These early‐life microbial interactions appear to play an important role in shaping immune responses and may influence susceptibility to allergic and immune‐mediated diseases later in life. A multinational birth cohort study demonstrated that maternal engagement in farming activities and consumption of farm‐produced dairy products during pregnancy correlated with elevated levels of T helper 1‐type, IFN‐gamma and TNF‐alpha cytokines in cord blood [10]. Additionally, research by Schaub et al. [11] revealed that mothers residing in traditional farms exhibited increased counts and functionality of cord blood T‐reg cells, accompanied by lower T(H)2 cytokine secretion and reduced lymphocyte proliferation upon innate exposure. Similarly, maternal exposure to stables and farm animals was associated with enhanced expression of immune response genes (e.g., *TLR2*, *TLR4* and *CD14*) during pregnancy, offering protection against the development of atopic sensitization in infants [12]. Moreover, studies conducted in the United States illustrated that maternal pet exposure significantly elevated Th1 levels, correlating with decreased allergic sensitization in infants [13].

Animal model studies have further elucidated the mechanisms underlying these observations. Bacteria isolated from farm cowsheds, including *Acinetobacter lwoffii* and *Lactococcus lactis*, have been found to confer protection against allergic airway inflammation by promoting a Th1 response [14, 15]. In addition, exposure to pets during pregnancy, be it cats or dogs, has been linked to reduced cord blood IgE levels and a diminished risk of atopy in infants [16]. Furthermore, maternal consumption of microbial‐rich farm‐produced dairy products or unpasteurised milk has been associated with altered cord blood cytokines, reduced total IgE levels and a shift from Th2 to Th1 immunity, thereby shielding offspring from asthma and allergy [10, 17].

Moreover, prenatal supplementation with probiotics such as *rhamnosus* or *Bifidobacterium lactis* has shown promise in modulating cord blood cytokines and enhancing immunoprotective components in breast milk [18]. Additionally, studies in murine models have indicated that a gluten‐free diet during pregnancy and lactation can modulate offspring immunity, resulting in a reduced proinflammatory immunological milieu in the gut and pancreas [19].

**Figure 1:**
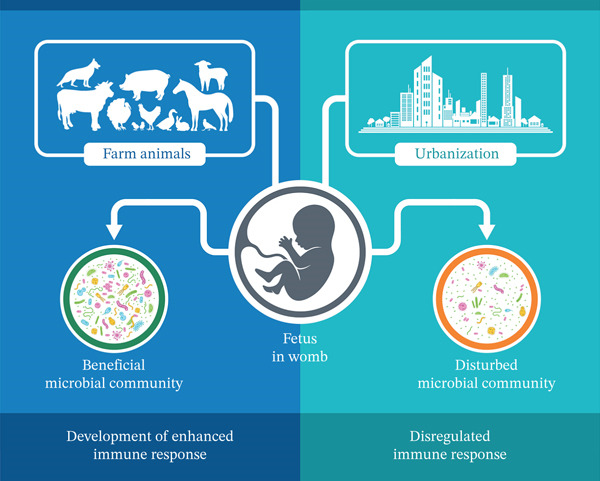
Maternal exposure to diverse environments influences the subsequent immune response of their children. In contrast to mothers in urban settings, those engaged in farm activities and consuming farm‐produced dairy products during pregnancy have been associated with offspring exhibiting an immune response that confers protection against allergies. (Note: Figure created by the authors based on information synthesized from the cited literature. Original artwork not reproduced or adapted from copyrighted sources.)

Collectively, these findings underscore the substantial impact of prenatal exposure to microbial compounds on fetal innate immune responses, with far‐reaching implications for the development of a child′s adaptive immune system (as depicted in Figure 1). Future research efforts should focus on exploring the diversity and functional correlations of maternal–fetal interface microbiota to pave the way for the development of probiotic and prebiotic therapies aimed at modulating offspring immunity.

## 3. Postnatal Microbiota: Influences of Delivery Mode, Feeding and Maturation

**Figure 2:**
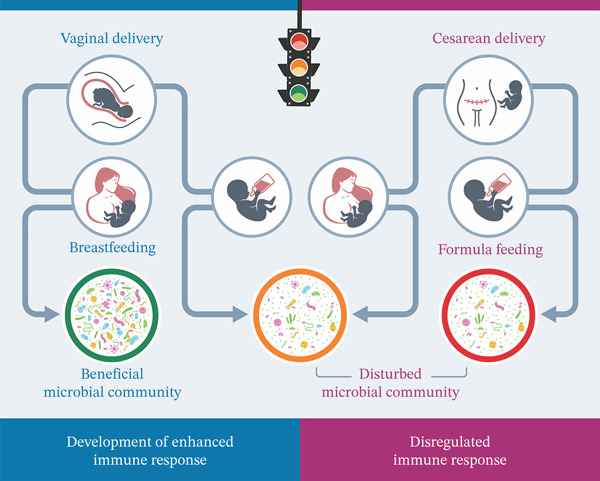
The method of delivery and early‐life feeding have significant implications for the development of the immune response. Children born through vaginal delivery are exposed to a high diversity of microorganisms, triggering a protective immune response. In contrast, those born through caesarean section have a less diverse microbiome, concerning the further immune adaptation. Moreover, breast milk and the areolar skin contain bacteria that contribute to a healthy immune response. Ideally, a child born through vaginal delivery and consuming breast milk would have the best immune response, whereas the opposite scenario would be a child born through caesarean section and consuming formula. However, children with less drastic alterations in microorganism exposure and immune response can be seen in other scenarios. (Note: Figure created by the authors based on information synthesized from the cited literature. Original artwork not reproduced or adapted from copyrighted sources.)

Originally, it was thought that postnatal microbial colonisation was highly influenced by the mode of delivery (see Figure 2). However, later studies suggest that age and gut maturation are more important factors in shaping the neonate microbiota [20, 21].

Notably, infants delivered vaginally exhibit a higher richness of *Bifidobacterium* species and lower levels of potentially pathogenic microbes such as *Enterobacter*, *Enterococcus* and *Klebsiella* compared with those born via caesarean section (C‐section) [22, 23]. C‐section‐born infants, on the other hand, display elevated abundance of oral and skin commensal bacteria, including *Corynebacterium*, *Staphylococcus*, *Streptococcus* and *Propionibacterium* species [24, 25].

Studies have shown that infants delivered by C‐section have lower microbial diversity and delayed or absent colonisation with fibre‐fermenting bacteria during the first 2 years of life. This delayed gut microbiome maturation can lead to developmental delays in the gut epithelium, mucus layer and T‐regulatory cells, for instance, which may increase susceptibility to allergic diseases later in life [23, 26].

Furthermore, breastfed infants have a significantly different microbiota composition than formula‐fed infants, and these differences are amplified in adulthood. Therefore, breastfeeding also promotes lifelong intestinal homeostasis [27]. Breastfed infants acquire bacteria such as *Bifidobacterium* species from breast milk, which are capable of metabolising human milk oligosaccharides (HMOs) [25, 28]. The younger neonate gut microbiome is enriched with lactate‐metabolised genes generally available in lactobacilli [29]. Strain‐specific metabolism of different types of HMOs in breast milk by *Bifidobacterium* promotes colonisation of specific *Bifidobacterium* species among different neonates [30].

The bacteria *Staphylococcus*, *Streptococcus* and *Lactococcus* are more predominant in human breast milk, and this composition differs drastically between mothers who underwent caesarean delivery and those who delivered vaginally. The composition also changes during the lactation period [31, 32]. Moreover, infants acquire 27% of their gut microbiota through maternal milk and an additional 10% from the areolar skin during the first year of life [33]. The shift from early infant feeding to solid food is associated with increased diversity and abundance of fibre‐fermenting *Firmicutes* and *Bacteroides*, as well as more short‐chain fatty acid metabolites [29, 34].

Postnatal microbiome is dynamically changing through life, intricately intertwining with our surroundings and the individuals in close proximity. A recent study found that people share their gut microbiota with their family members and cohabitants. Young children may have up to 50% of similar bacteria to their mothers, and unrelated cohabitants have between 12% and 32% microbiota similarity [35]. Therefore, these findings suggest that the gut microbiota is influenced by the social environment and the individuals with whom we share our habitat.

Thus, the postnatal microbiota is shaped by the delivery mode, age, gut maturation, feeding practices and social interaction. Understanding these multifactorial influences is paramount, as they not only influence the neonatal microbiota composition but also impact the long‐term health and immune responses of the individual. Further research in this field holds the key to unravelling the intricate dynamics of postnatal microbial colonisation.

## 4. The Interaction Between the Microbiome and Inflammatory Bowel Disease (IBD)

Mounting evidence underscores the pivotal role of the gut microbiome, comprising both bacteria and fungi, in the intricate pathogenesis of IBDs, encompassing ulcerative colitis (UC) and Crohn′s disease (CD). Extensive research has illuminated the contribution of factors such as diminished microbial diversity and the depletion of specific bacterial clades, notably Enterobacteriaceae, in the onset of IBD [36–38].

Dysbiotic expansion of Enterobacteriaceae, particularly observed in new‐onset CD, has been precisely targeted in mouse models, leading to amelioration of CD severity [39]. Additionally, the depletion of symbiotic bacteria like *Faecalibacterium prausnitzii* in IBD patients has been correlated with recurrent CD, yet supplementation of these bacteria counterbalances dysbiosis, displaying potent anti‐inflammatory effects in murine models [40]. Moreover, research has identified a 15‐KDa anti‐inflammatory protein in *F. prausnitzii* culture supernatant, which effectively reduces intestinal inflammation by inhibiting the NF‐*κ*B pathway [41, 42].

Several studies showed that helminth colonisation potentially regulates intestinal inflammation in IBD [43]. Helminth colonisation has been shown to diminish inflammation by promoting the expansion of anti‐inflammatory *Clostridiales*, inhibiting proinflammatory bacterial taxa in genetically predisposed IBD mice [44]. It is noteworthy, however, that the effectiveness of helminth introduction in ameliorating IBD seems to be contingent on the presence of specific genetic variants, with clinical trials showing variable outcomes in patients lacking predisposing genetic markers for CD.

It is crucial to understand the microbiome changes in IBD patients. Further clinical trials are essential to elucidate the impact of microbiota and dysbiosis on these conditions. Exploring ways to restore the balance of appropriate microorganisms holds the key to effectively managing this intricate gastrointestinal and immunological disorder.

## 5. The Microbiome and Cardiovascular Diseases

Cardiovascular diseases loom large as major contributors to global morbidity and mortality [45]. Recent studies have spotlighted the pivotal role of gut microbiota dysbiosis in the intricate pathophysiology of cardiovascular diseases. Notable research findings have revealed a compelling connection between specific microbial compositions and various cardiovascular conditions. In a case–control study focused on coronary heart disease (CHD), a decreased proportion of the Bacteroidetes phylum and an increased proportion of *Firmicutes* were noted in CHD patients compared with controls, underscoring the significance of these bacterial clades [46]. Similarly, in atherosclerotic cardiovascular disease (ACVD), patients exhibited an elevated abundance of Enterobacteriaceae and *Streptococcus* spp., coupled with reduced *F. prausnitzii* and *Roseburia intestinalis*, characterising a gut microbiome profile marked by inflammation [47]. The dysregulated microbiota in patients with cardiovascular diseases also extends to conditions like coronary artery disease (CAD) and atherosclerosis, where an imbalance of *Enterococcus*, *Escherichia*–*Shigella*, *Faecalibacterium*, *Roseburia*, *Subdoligranulum* and *Eubacterium* has been observed, linking microbial dysbiosis with metabolic dysfunction [48].

Furthermore, studies have pinpointed specific genetic factors and dietary components influencing the gut microbiota. Notably, genes encoding peptidoglycan synthesis were found to be enriched in ACVD patients, and a robust negative correlation was identified between C‐reactive protein (CRP) levels and the gut microbiota of healthy controls, hinting at the immune‐inflammatory interplay within the microbiome [49]. Additionally, the Western diet, rich in choline and carnitine, significantly amplifies cardiovascular risk [50, 51], a phenomenon linked to the production of trimethylamine‐N‐oxide (TMAO) via hepatic flavin‐containing monooxygenases. This TMAO, identified as a potent predictor of CAD, showcases the relationship between dietary patterns, microbial metabolism and cardiovascular health [52–55].

Intriguingly, innovative animal studies have explored the potential of microbial interventions. Targeted inhibition of trimethylamine (TMA) generation from gut microbiota resulted in decreased atherosclerotic plaque development in susceptible *Apoe* ^−/−^ mice, hinting at a promising avenue for future therapeutics [56]. Notably, in a Western diet–fed animal model, supplementation with *A. muciniphila* in drinking water counteracted the atherosclerosis‐promoting effects of TMAO precursor, underscoring the potential of microbial modulation as a preventive strategy [57].

The microbiome composition also varies in people with cardiovascular risk factors, with obesity or diabetes. Individuals with obesity often exhibit distinct gut microbiota patterns, characterised by decreased bacterial diversity and an imbalance in specific bacterial groups [58]. This altered microbiota composition can affect the body′s energy balance and fat storage mechanisms. For instance, certain gut bacteria are known to extract and store more energy from the diet, contributing to weight gain [59]. The microbiome disruption influences inflammation and metabolic dysregulation linked to obesity [60]. These findings underscore the potential of microbiome‐targeted interventions in managing and preventing obesity.

The relationship between the microbiome and diabetes is an area of growing interest. Research indicates that individuals with diabetes often exhibit gut microbiota imbalances, characterised by reduced diversity and altered compositions [61]. For example, a reduction in butyrate‐producing bacteria and an increase in opportunistic pathogens are commonly observed [62]. These microbial changes can contribute to insulin resistance and glucose intolerance, key features of diabetes. Thus, the gut microbiota influences the body′s inflammatory responses and metabolic pathways as long as diabetes progresses [63].

The future of cardiovascular disease and its risk factors prevention lies in the nuanced understanding and modulation of microbial metabolism through direct supplementation or dietary interventions, offering a tantalising prospect for novel therapeutic approaches.

## 6. Microbiome and Obesity

The prevalence of obesity in the world is increasing in the last years. Diet, lifestyle and genetic factors are related to the development of obesity. If we can modify these factors, we could potentially facilitate getting back to a normal body weight. Gut microbiota has received special attention in the last years since restrictive diets and bariatric surgery produce changes in the microbial composition. Prebiotics have been shown to restore a healthy microbiome and reduce body fat [64, 65]. It has also been observed that the gut microbiota is different in healthy weight as compared with obese individuals [66]. Intermittent fasting is also promising for weight loss, which has been shown to alter the gut microbiota [67]. Manipulation of the intestinal microbiota has been linked to weight changes and obesity [68].

Recent studies have shown that the development of obesity may be influenced by shifts in gut microbiota in response to the intake of fat in diet. Furthermore, these alterations in gut microbiota have been shown to promote important changes in satiation signals that promote appetite and therefore obesity [69].

Targeting gut microbiota with synbiotics (probiotic supplements containing prebiotic components) is emerging as a promising intervention in the comprehensive nutritional approach to reducing obesity. Weight loss resulting from low‐carbohydrate high‐protein diets can be significant but has also been linked to potentially negative health effects due to increased bacterial fermentation of undigested protein within the colon and subsequent changes in gut microbiota composition. Correcting obesity‐induced disruption of gut microbiota with synbiotics can be more effective than supplementation with probiotics alone because the prebiotic components of synbiotics support the growth and survival of healthy bacteria [66, 70].

## 7. The Interplay Between Microbiome and Autoimmunity

Over the past several decades, the incidence of autoimmune conditions has been increasing [71, 72]. A crucial aspect of autoimmune tissue damage involves molecular mimicry of human antigens, potentially orchestrated by immune system reprogramming influenced by gut microbiota [73]. Case–control studies examining Type 1 diabetes mellitus (T1DM) have revealed a notable reduction in the *Firmicutes*/*Bacteroides* (F/B) ratio among patients compared with healthy subjects, indicating a microbial imbalance [74–76]. Initially, studies showed decreased microbial diversity in both T1DM [77] and seroconverted children [78, 79], but this reduction was specific to seroconverts rather than undeveloped seroconverters [80]. For instance, infants from regions where T1DM is prevalent, such as Finland and Estonia, exhibited enriched gut microbiota in *Bacteroides* spp., mainly with *Bacteroides dorei*. This enrichment allowed their primary LPS exposures from *Bacteroides* rather than *E. coli*, which has a structurally distinct LPS. In agreement with this, an animal model study demonstrated that primary exposure to *B. dorei* LPS inhibits innate immune signalling and increases susceptibility to T1DM [81].

Rheumatoid arthritis (RA), a chronic inflammatory disease–causing joint destruction, has also been linked to gut microbiota. Studies have identified an increased abundance of *Prevotella copri* in new‐onset untreated rheumatoid arthritis (NORA) patients, along with reduced *Bacteroides*, compared with healthy controls [82]. Recently, European population–based research further confirmed the rich colonisation of *Prevotella* species, including *P. copri*, in preclinical stage RA patients compared with controls [83]. Animal models, specifically germ‐free arthritis‐prone SKG mice inoculated with faecal samples from early RA patients, developed T cell–mediated arthritis, emphasising the role of dysbiotic microbiota in activating innate immunity [84]. Another molecular study revealed high homology between RA‐specific autoantigens and *Prevotella*‐associated peptides, highlighting a potential link between immune responses to microbial peptides and host autoimmune responses [85]. However, further research is imperative to understand the precise editing of gut microbiota and the immune responses in RA patients, offering promising avenues for targeted therapeutic interventions.

## 8. The Connection Between Microbiome and Cancer

Over the past 15 years, a myriad of studies has unveiled intricate connections between host microbiota and both physiological and pathophysiological processes. These investigations highlight the microbiota′s significant role in cancer development and its associations with various pathological conditions, including neurological, cardiovascular and gastrointestinal disorders [86, 87].

The collective evidence suggests that distinct microbial communities within the gut wield influence over cancer development by modulating inflammation, immune responses and the metabolism of dietary components [88]. Dysbiosis has been linked to an elevated susceptibility to diverse cancers, including colorectal cancer, hepatocellular carcinoma and pancreatic cancer [89, 90].

The microbiome influences cancer development through diverse pathways. The dynamic interplay between the host immune system and pathogenic microbes orchestrates chronic inflammation, fostering a procarcinogenic microenvironment. Additionally, microbially produced metabolites, including short‐chain fatty acids and secondary bile acids, play a role in modulating cellular processes associated with cancer risk [91].

Beyond its role as a contributor to cancer pathology, the microbiome also holds promise as a diagnostic tool. Recent studies have explored cancer‐specific microbial patterns in biofluids, such as blood and faeces, offering noninvasive avenues for early cancer diagnosis and risk stratification [92]. Advances in metagenomic and metabolomic techniques contribute significantly to the identification of these microbial markers.

Moreover, recent investigations underscore the potential influence of the gut microbiota on the efficacy of various cancer immunotherapies [93, 94]. Notably, cancer patients who responded positively to anti–programmed cell death protein 1 (PD1) therapy exhibited a highly diverse and rich gut microbiota, in contrast to nonresponders characterised by consistently low diversity and a high relative abundance of *Bacteroidetes* [95–97]. Faecal microbiota transplantation (FMT) from treatment responders or supplementation with bacterial taxa depleted in nonresponders into mice enhanced the efficacy of anti‐PD1 treatment, accompanied by improved antitumor T cell responses [93, 96].

Furthermore, metagenomic and metabolomic profiling studies have identified significant differences between the intestinal metabolites of immune checkpoint inhibitor (ICI) responders and the disease‐promoting population, highlighting the composition of the gut microbiota as a determinant of host responses to ICIs [98]. In this sense, Vetizou et al. [99] demonstrated in their work that tumours in germ‐free or antibiotic‐treated mice failed to respond to CTLA blockade. The observed immunostimulatory effect was intricately linked to specific T cell responses triggered by distinct *Bacteroides* species. Therefore, manipulating the interaction between host and microbiota holds promise as a viable strategy to overcome resistance to ICIs, thereby enhancing the efficacy of immune therapies [97].

The interplay between the gut microbiota and adoptive T cell therapies, particularly chimeric antigen receptor (CAR) T cell therapy, introduces complexity to advanced oncologic therapies. Murine models reveal the microbiota′s role in orchestrating systemic immune dynamics, shaping administered T cell functionality and persistence [100]. This understanding is crucial for refining treatment precision and advancing our knowledge of the microbiome′s pivotal role in cancer immunotherapy. These revelations open avenues for bespoke therapeutic interventions at the intersection of microbiome research and modern cancer therapeutics [101].

Hence, exploring the microbiome′s involvement in cancer could elucidate the disease′s onset in the presence of dysbiosis and pave the way for employing biomarkers in personalised oncology for diagnosis, stratification and treatment.

## 9. Transplantation of Beneficial Microbiota for Restoring Dysbiosis and Improve Diseases

FMT has demonstrated a cure rate exceeding 90% in the treatment of recurrent antibiotic‐resistant *Clostridium difficile* infection [102]. These findings have stimulated the evaluation of FMT as a therapeutic strategy for a broad range of gastrointestinal and extraintestinal disorders associated with microbial dysbiosis. Despite increasing clinical interest, the therapeutic efficacy of FMT in IBD remains incompletely understood, and the preliminary clinical trial results are contradictory and limited. However, implementation of modified strategies like intensive dosing, multidonor FMT use in IBD patients has been shown to improve clinical outcomes [103, 104].

Several open‐label trials with small cohorts and meta‐analysis have shown the application of FMT in irritable bowel syndrome has produced effective treatment results [105–108]. FMT has been implemented in chronic inflammatory conditions affecting organs distal to the GI tract. An open‐label clinical trial of 18 autistic children showed the impact of FMT altered the gut ecosystem and improved gastrointestinal and autism symptoms. These improvements were sustained 8 weeks after treatment [109].

In another study, improved insulin sensitivity was observed in obese and metabolic syndrome men treated with FMT from lean donors and this improvement was associated with improved plasma metabolites, in particular increased phenylalanine, gamma‐aminobutyric acid (GABA) and tryptophan [110]. On the other hand, FMT from lean vegan donors to male metabolic syndrome patients resulted in changed gut microbiota composition toward a vegan profile without altering the capacity of TMAO production or parameters of vascular inflammation [111].

Several case reports and animal models have also demonstrated significant positive therapeutic effects of FMT in conditions such as multidrug‐resistant organisms (MDRO) infections [112], severe multiple sclerosis [113] and multiple organ dysfunction in critical patients [114]. On the other hand, FMT from cancer patients responding to anti‐PD1 treatment into germ‐free mice enhanced the effectiveness of anti‐PD1 treatment, leading to heightened antitumor T cell responses [93]. Nevertheless, FMT is not without risks. A recent study revealed the transmission of drug‐resistant *E. coli* bacteremia in two patients from a donor who had undergone FMT, resulting in the unfortunate demise of one patient [115].

Recently, the food and drug administration (FDA) approved two FMT products with safety and efficacy demonstration for the treatment of recurrent infections with *C. difficile*. The first of this mode was REBYOTA, whose safety [116, 117] and efficacy [117] even farther than 8 weeks has been reported. Moreover, this product seems to improve the quality of life of patients receiving this product during the treatment of their pathologies [118], and seems to represent a cost‐effective approach for previous recalcitrant—and without any other alternative—patients; thus, helping the healthcare system.

Conversely to the rectal administration of REBYOTA, a new oral product has been approved by the FDA. Vowst represents the first oral product as a FMT approach and has proven clinical improvement at Week 8 in as high as 91.3% of the patients enrolled in the ECOSPOR‐IV clinical trial, the last version of the original trials involving Vowst [119]. Real‐world analysis could help to better understand the effect of this oral FMT strategy and further similar strategies that could emerge.

**Figure 3:**
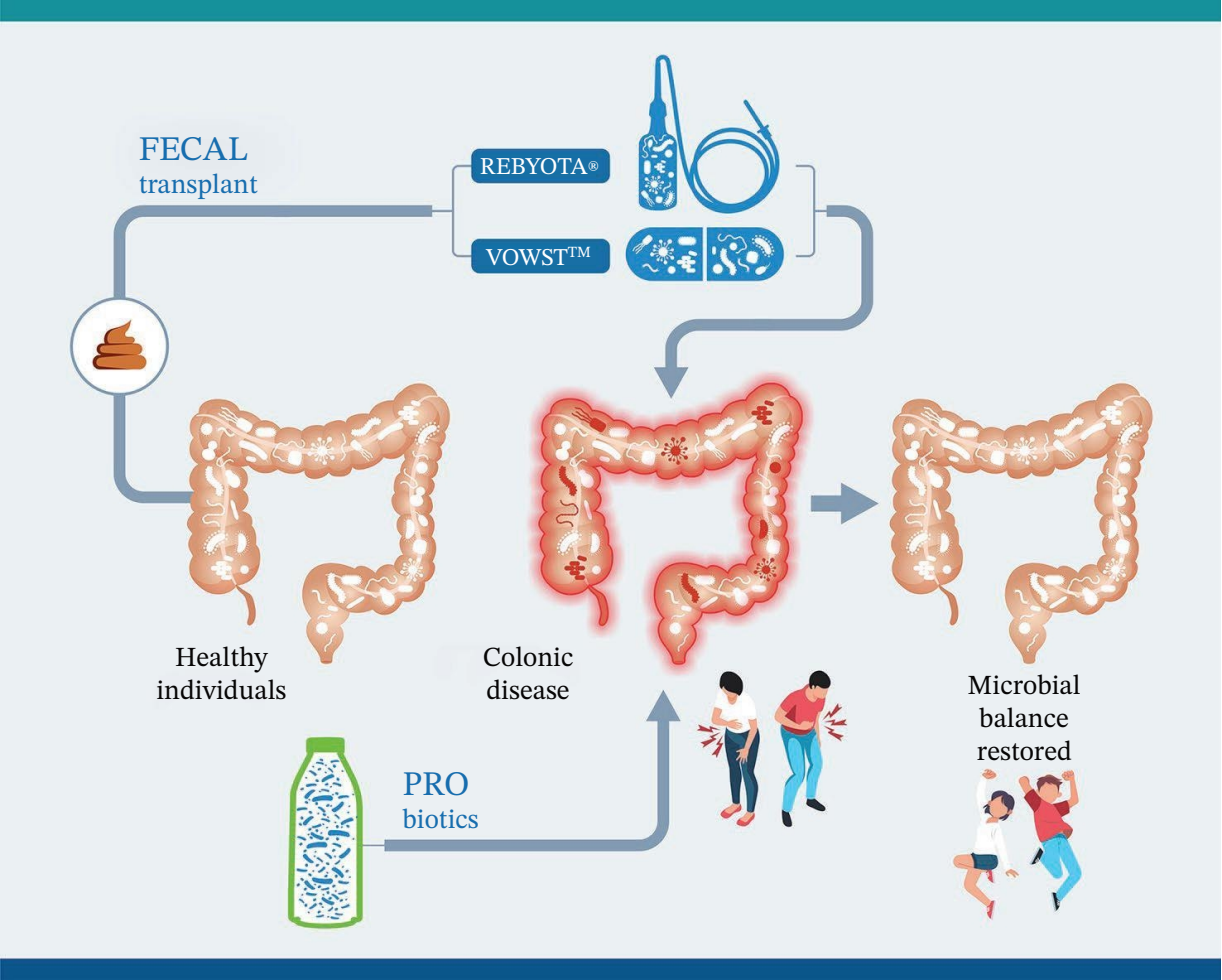
Current and emerging applications of faecal microbiota transplantation (FMT). The application of FMT to treat conditions such as recurrent *Clostridium difficile* infections and inflammatory bowel disease is noteworthy. The introduction of protective bacteria is believed to restore the disrupted microbiota in these patients. Clinical trials for the development of REBYOTA and Vowst have exhibited considerable efficacy in addressing these conditions. The administration routes for REBYOTA and Vowst are rectal and oral, respectively. The use of probiotics is also considered to have the potential to restore or help balance the intestinal microbiota. (Note: Figure created by the authors based on information synthesized from the cited literature. Original artwork not reproduced or adapted from copyrighted sources.)

FMT is proving the clinically evident safety and effectiveness for management or prevention of certain disorders but there is a need to standardise and optimise procedures like screening for suitable donors and personalise treatments according to each patient′s dysbiosis (see Figure 3). Despite the current FDA FMT‐approved products, price is still high for low resource countries, and further developments with more accessibility will be globally ground breaking.

## 10. The Use of Probiotics to Promote a Healthy Microbiota

Recent studies have highlighted the critical influence of the composition of autochthonous gut microbial communities on the successful colonisation ability and persistence effects of allochthonous microbes [120, 121]. Surprisingly, this interaction factor has been largely overlooked in microbial interference–based clinical trials, possibly contributing to inconsistencies observed between animal model studies and human trials.

Nevertheless, multiple investigations have demonstrated that the administration of a multispecies probiotic preparation (VSL#3) can induce effective clinical responses and remissions in patients with recurrent UC [122, 123]. This positive effect has not been replicated in CD patients [124].

Moreover, significant clinical improvements have been associated with reduced insulin resistance, body mass index and increased glucagon‐like peptide levels in children with nonalcoholic fatty liver disease following 4 months of daily supplementation with the same probiotic preparation (VSL#3) [125]. In the context of Type 2 diabetes mellitus (T2DM), a meta‐analysis of randomised controlled trials revealed moderate improvements in hyperglycaemia with interventions using various combinations of bacterial strains in probiotics [126]. Additionally, a large multicentre prospective cohort study demonstrated a reduced risk of islet autoimmunity in children with a high genetic risk for T1DM who were supplemented with probiotics during the first 27 days of life, compared with no supplementation or supplementation later in infancy [127].

Furthermore, a meta‐analysis of randomised, placebo‐controlled trials indicated a reduced risk of atopic sensitization and decreased total IgE levels in children with prenatal and/or early‐life probiotic interventions [128]. A follow‐up of a randomised, double‐blind, placebo‐controlled trial revealed that 67% of children who received combined probiotic (*Lactobacillus rhamnosus*) and peanut oral immunotherapy (PPOIT) for 18 months remained desensitised to peanuts after 4 years, compared with 4% in the placebo group [129]. However, the study was unable to pinpoint whether the observed beneficial effect was attributable to the immunotherapy, the probiotic, or both due to the lack of individual component control groups. Despite this limitation, the results are encouraging and suggest long‐term efficacy in preventing food allergies with the combined administration of probiotic and PPOIT.

Nevertheless, early‐life oral microbial intervention trials that showed significant positive results for atopic sensitization have failed to demonstrate protection against asthma or wheeze [128]. A microbial (*L. rhamnosus* GG–LGG) intervention during the first 6 months of high‐risk infants for asthma failed to prevent the development of asthma by 2 years of age [130].

Conversely, a subsequent study demonstrated that early‐life LGG supplementation in high‐risk infants prevented delayed gut microbiota bacterial diversification and metabolic dysfunction. Moreover, ex vivo LGG intervention in faecal microbes was associated with increased T‐reg cell count. Unfortunately, the colonisation of the gut with LGG and the associated metabolic reprogramming benefits were not sustained over an extended period [131].

In summary, although probiotic intervention studies have shown encouraging results, more attention is warranted in strain selection based on metabolic functional characteristics, defining supplementation duration and considering the interaction effects of allochthonous microbes on the recipient autochthonous gut microbial community. Addressing these factors may significantly enhance the efficacy of probiotics in future interventions.

## 11. The Neglected Microbiota: Indigenous Populations

Understanding the microbiome of indigenous populations is crucial for drawing the complete story of the human microbiome. These neglected populations have unique microbial profiles shaped by distinct environmental and lifestyle factors compared with nonindigenous counterparts, with changes in the composition and diversity of faecal, oral or skin microbiota [132–134].

Research of microbiome composition in indigenous populations can help us to understand the human susceptibility to harbour specific genes related with antibiotic resistance (resistomes) even without antibiotics usage [134]. For example, the oral microbiota of the Temuan people, an urbanised group, showed a higher presence of opportunistic pathogens like *Corynebacterium*, *Prevotella* and *Mogibacterium*, indicating potential oral imbalances [135]. On the other hand, the Yanomami Amerindian population showed a high diversity and abundance of microbial composition in faecal and skin samples, compared with urbanised people. This could be explained by the changes and diversity of meals seasonality and lack of clothing. Notably, intestinal *Oxalobacter* and *Desulfovibrio* were significantly increased in the Yanomami compared with U.S. subjects, whereas the oral microbiome exhibited similarities between the indigenous population and urbanised people [134].

Beyond genetic and cultural factors, socioeconomic determinants such as sanitation, dietary quality, urbanisation, antibiotic exposure and access to healthcare play a fundamental role in shaping the human microbiota and may contribute to population‐level health disparities.

Urbanisation and Westernised dietary patterns—characterised by high intake of ultraprocessed foods, low fibre consumption and increased antibiotic use—have been consistently linked to reduced gut microbial diversity and loss of ancestral microbial taxa [136–138]. In contrast, rural and low‐industrialised populations often exhibit higher microbial richness and distinct functional metabolic pathways, largely driven by fibre‐rich diets and greater environmental microbial exposure [139].

Improvements in sanitation and reduced exposure to environmental microbes, while essential for preventing infectious diseases, have also been associated with decreased microbial diversity and altered immune education, consistent with refinements of the hygiene hypothesis [135]. However, limited healthcare access and inadequate sanitation in low‐resource settings may increase susceptibility to enteric infections and chronic inflammation, contributing to the double burden of infectious and noncommunicable diseases [140].

Collectively, these findings indicate that socioeconomic context acts as a structural determinant of microbiome configuration, potentially mediating disparities in obesity, autoimmune diseases, metabolic disorders and allergic conditions across populations. Addressing these determinants through scalable public health strategies—including maternal nutrition programs, rational antibiotic stewardship, improved sanitation infrastructure and breastfeeding promotion—may represent cost‐effective approaches to modulate microbiome‐associated disease risk at the population level.

Thus, including indigenous and uncontacted populations can help to write the humankind microbiome history. Investigating the gut microbiomes of hunter‐gatherers and traditional societies offers a chance to identify ‘ancestral’ microbiome characteristics and explore their role in human evolution and this requires efforts to map the largely unexplored microbiome diversity in these populations, employing both culture‐based techniques and genomic reconstruction methods [141].

## 12. Limitations and Future Perspectives

This narrative review has several limitations that warrant careful consideration. First, as a nonsystematic review, study selection may be influenced by publication bias and does not follow a structured framework for evidence grading. Although narrative approaches allow for broad thematic integration, they inherently limit reproducibility and quantitative synthesis of findings.

Second, a substantial proportion of microbiome research is based on observational and cross‐sectional studies, as well as preclinical animal models. Although these designs are valuable for hypothesis generation and mechanistic exploration, they restrict causal inference. Associations between microbial composition and disease states may reflect reverse causation or residual confounding factors—including diet, medication use, socioeconomic conditions, environmental exposures and host genetics—rather than direct mechanistic effects [142].

Importantly, not all reported microbiome–disease associations are consistently replicated across studies or populations. Several large‐scale analyses have demonstrated attenuation or loss of statistical significance after adjusting for confounding variables [142]. Microbial taxa initially identified as disease‐associated in one cohort are not always reproducibly observed in independent validation studies, underscoring the dynamic and context‐specific nature of host–microbiome relationships [143]. Similarly, interventional trials—including probiotic supplementation, dietary modulation strategies and other microbiota‐targeted approaches—have shown heterogeneous or modest effects influenced by strain specificity, host characteristics, baseline microbial composition and study design [144]. Consequently, caution is required when interpreting microbiome–disease correlations and when translating such findings into clinical practice or public health recommendations.

Methodological heterogeneity further complicates interpretation. Variability in sample collection (e.g., stool vs. mucosal biopsies), storage conditions, DNA extraction protocols, sequencing platforms (16S rRNA gene sequencing versus whole metagenomic shotgun sequencing) and bioinformatic pipelines can substantially influence taxonomic and functional profiles [145]. Differences in statistical modelling, normalisation strategies, reference databases and reporting standards contribute to limited cross‐study comparability and reproducibility. Greater methodological standardisation and transparent reporting practices are therefore essential to strengthen the robustness of microbiome research.

Moreover, key conceptual terms require clarification. The term ‘dysbiosis,’ although widely used to describe disease‐associated alterations in microbial communities, lacks a universally accepted definition. Rather than representing a single pathological microbial signature, dysbiosis reflects context‐dependent shifts in microbial diversity, relative abundance or functional capacity that vary across populations, disease states and environmental contexts [146, 147]. Overreliance on compositional descriptors without functional or mechanistic validation risks oversimplifying complex host–microbiome interactions.

Although microbiota‐targeted interventions have generated considerable enthusiasm, most clinical trials have been conducted in highly controlled environments and predominantly within high‐income countries. Differences in diet, sanitation, infectious disease burden, antibiotic exposure, healthcare infrastructure and baseline microbiota composition may substantially influence both efficacy and safety in other settings. Therefore, caution is warranted when extrapolating findings beyond the populations in which they were originally studied. The scalability, cost‐effectiveness, cultural acceptability and long‐term sustainability of microbiome‐based strategies must also be critically evaluated before broad implementation in public health frameworks.

Future research should prioritise large‐scale longitudinal cohort studies and rigorously designed randomised controlled trials to better define causal relationships between microbiota composition, functional activity and disease outcomes. Integration of multiomics approaches—including metagenomics, metatranscriptomics, metabolomics and proteomics—will be crucial to advance mechanistic understanding beyond taxonomic descriptions. Standardisation of methodologies and harmonisation of analytical frameworks will further enhance reproducibility. Additionally, expanding research to include underrepresented, rural and indigenous populations is essential to capture global microbiome diversity and to ensure equitable development of microbiome‐based diagnostics, preventive strategies and therapeutics. Only through methodological rigor, conceptual clarity, contextual sensitivity and inclusive research frameworks can microbiome science be responsibly translated into meaningful and globally applicable clinical and public health impact.

## 13. Conclusions

This paper highlights the intricate role and changes of the human microbiota from prenatal stages to adult health, with some examples of the microbiome research arena in understanding pathologies. The growing understanding of host–microbiome interactions is opening new avenues for biomarker discovery, precision diagnostics and microbiota‐targeted therapeutics as we uncover the complexities of these microbial ecosystems, their profound implications for health, disease and medicine become increasingly evident, steering us toward a future where microbiome research is integral to advancing human health and precision medicine globally.

## Author Contributions

Heinner Guio was the project leader of the review. Heinner Guio designed the review. Julio A. Poterico and Babu G. Venkatesh summarized and described the review findings. Luis Jaramillo‐Valverde, César Sanchez, Erick Alayo and Julio Valdivia‐Silva interpreted and discussed review findings. Julio A. Poterico prepared the figures. All prepared the final version.

## Funding

No funding was received for this manuscript.

## Conflicts of Interest

The authors declare no conflicts of interest.

## Data Availability

The data that support the findings of this study are available from the corresponding author upon reasonable request.
